# Real time 3D monitoring of sintering ore temperature enabled by temporal fusion transformers

**DOI:** 10.1038/s41598-025-27254-9

**Published:** 2025-12-09

**Authors:** Yushan Jiang, Yifei Wang, Tiantian Ma, Huan Yang, Zhaoxia Wu, Haoyi Zhao, Yunxiao Chang, Xuanyu Shen, Xinjun Cai, Yuxin Wu, Ruiyu Xie

**Affiliations:** 1 School of Mathematics and Statistics, Northeastern University at Qinhuangdao, Qinhuangdao, China; 2 Institute of Data Analysis and Intelligent Computing, Northeastern University, Shenyang, China; 3 School of Control Engineering, Northeastern University, Shenyang, China; 4 State Key Laboratory of Integrated Automation of Process Industry, Northeastern University, Shenyang, China

**Keywords:** 3D monitoring of sinter ore temperature, TFT, Interpretability, Chemical composition, VAE-TCN, Fossil fuels, Scientific data, Electrical and electronic engineering, Information technology, Applied mathematics

## Abstract

Iron ore serves as the fundamental feedstock for blast furnace operations, and its quality is constitutionally affected by the temperature of the mixture during the sintering process. To enhance the early prediction and regulation of the mixture temperature, this study proposes an intelligent 3D prediction model for iron ore temperature based on the Temporal Fusion Transformer (TFT). This model effectively combines advanced multi-horizon forecasting capabilities with temporal dynamic interpretability, while expanding the observation framework into a three-dimensional space through simulation outcomes. Simultaneously, the study focuses on the fluctuation patterns of the major chemical components in sintering materials and their influence on iron ore temperature through the Variational Autoencoder-Temporal Convolutional Networks (VAE-TCN) model. The TFT model, developed using historical sintering data, achieves an $R^{2} = 0.8572$ and RMSE = 4.7568 for one-step-ahead prediction of the sinter temperature spatial distribution, based on a dataset split of 90% training, 5% validation, and 5% testing. Compared with Transformer and Long Short-Term Memory (LSTM) networks, the TFT model demonstrates superior performance, reducing RMSE by 0.805 and 2.9937, respectively. In practical applications, the TFT model offers valuable guidance for real-time temperature monitoring during iron ore sintering operations.

## Introduction

According to the Implementation Guide for Digital Transformation in the Iron and Steel Industry emphasizing ”Promoting Intelligent Optimization of the Production Process” and the ”Guidelines for the Construction of an Intelligent Manufacturing Standard System in the Iron and Steel Industry (2023 Edition)” issued by the Ministry of Industry and Information Technology of the People’s Republic of China, the goal is to ”improve the numerical control rate of key processes,” and observation and intelligent adjustment of the sintering process has always been a long-term goal of the national steel industry^1^. Temperature distributions that are too high or too low can lead to the formation of overly cold or overly combustible materials, resulting in a lot of waste. Currently, the sintering process is based on methods such as infrared thermal imaging, making it difficult to accurately observe the internal state in real time. Therefore, it is essential to develop an algorithmic model to predict the internal temperature based on external data^2^.

The complexity of the sintering process, coupled with the interplay of multiple factors, makes precise monitoring the focus of the research on the ironmaking process. Some scholars have analyzed the reaction behavior between *C* and hematite, and found that the content of *C* and the reaction temperature have significant effects on the reaction rate and effective diffusion coefficient^3^. Some scholars have emphasized the key role of the ore components in the sintering process^4^. Other scholars emphasized the importance of content $SiO_2$ and *MgO* to ensure consistent iron ore performance and optimize production efficiency. However, these studies focus only on the main chemical components selected in the mixture^5^.

A thermochemical database was developed using the CALPHAD method, enabling the evaluation of iron ore behavior through phase equilibrium studies^6^. However, the study did not discuss the specific effects of the main chemical components on the temperature of iron ore during the sintering process. A one-dimensional mathematical model was proposed to predict the temperature distribution in thermally sintered materials^7^. This model accounted for factors such as the compressible flow of gas, the instabilities of heat conduction in solids, and the influence of the porosity of the bed on gas flow and heat transfer. However, the actual sintering process occurs in a three-dimensional space, which limits the applicability of the model. In addition, advances in data acquisition technologies were highlighted as key enablers for the adoption of machine learning modeling methods^8^. However, challenges persist in managing high-dimensional and dynamically changing data during computation. Furthermore, many researchers have turned their attention to deep learning models. For example, studies using Back Propagation (BP) neural networks have investigated the mapping relationship between process parameters and the chemical composition of iron ore. Despite these efforts, the interpretability of such ”black-box” models remains a significant area for improvement.

Recent years have witnessed growing applications of deep neural network architectures across diverse domains^9^. The Long Short-Term Memory (LSTM) model has found extensive use in time series forecasting. On the other hand, the Transformer model, with its superior long-term memory capabilities and non-linear mapping advantages, has demonstrated successful implementation in multiple domains including image recognition^10^, medical data processing^11,12^, and text processing^13,14^, among others^15^. In 2021, an improved model was proposed called Temporal Fusion Transformer (TFT)^16^, which substantially enhances the original Transformer architecture. By integrating components such as gated residual networks, variable selection networks, static covariate encoders, multi-head attention mechanisms, and the temporal fusion decoder, the TFT model is well suited for handling diverse input types, including static covariates. The TFT model effectively handles non-linear relationships in predictions, eliminates redundant components to improve performance, and captures long-term dependencies across various time steps. It also identifies key variables and important patterns while minimizing the contribution of irrelevant input, thereby improving the interpretability of the model. These features make it possible to focus on predicting the spatial temperature distribution within iron ore in this study.

Therefore, this research introduces an innovative 3D model for real-time prediction and monitoring of iron ore internal temperature, accompanied by comprehensive simulation results. The key contributions include:

- A novel 3D prediction model for iron ore temperature is proposed, extending the dimensional scope of temperature observation during sintering. By entering external data, including sensor information, the model predicts the temperature at various points within the iron ore and identifies its key factors of influence.
- The study focuses on analyzing the impact of the major chemical component ratios of the mixture, which are identified as key factors influencing iron ore temperature.
- The experimental research sintering process is simulated, and the effectiveness of the established 3D sinter temperature prediction model is verified.

## Mechanistic foundations for prediction

**Fig. 1:**
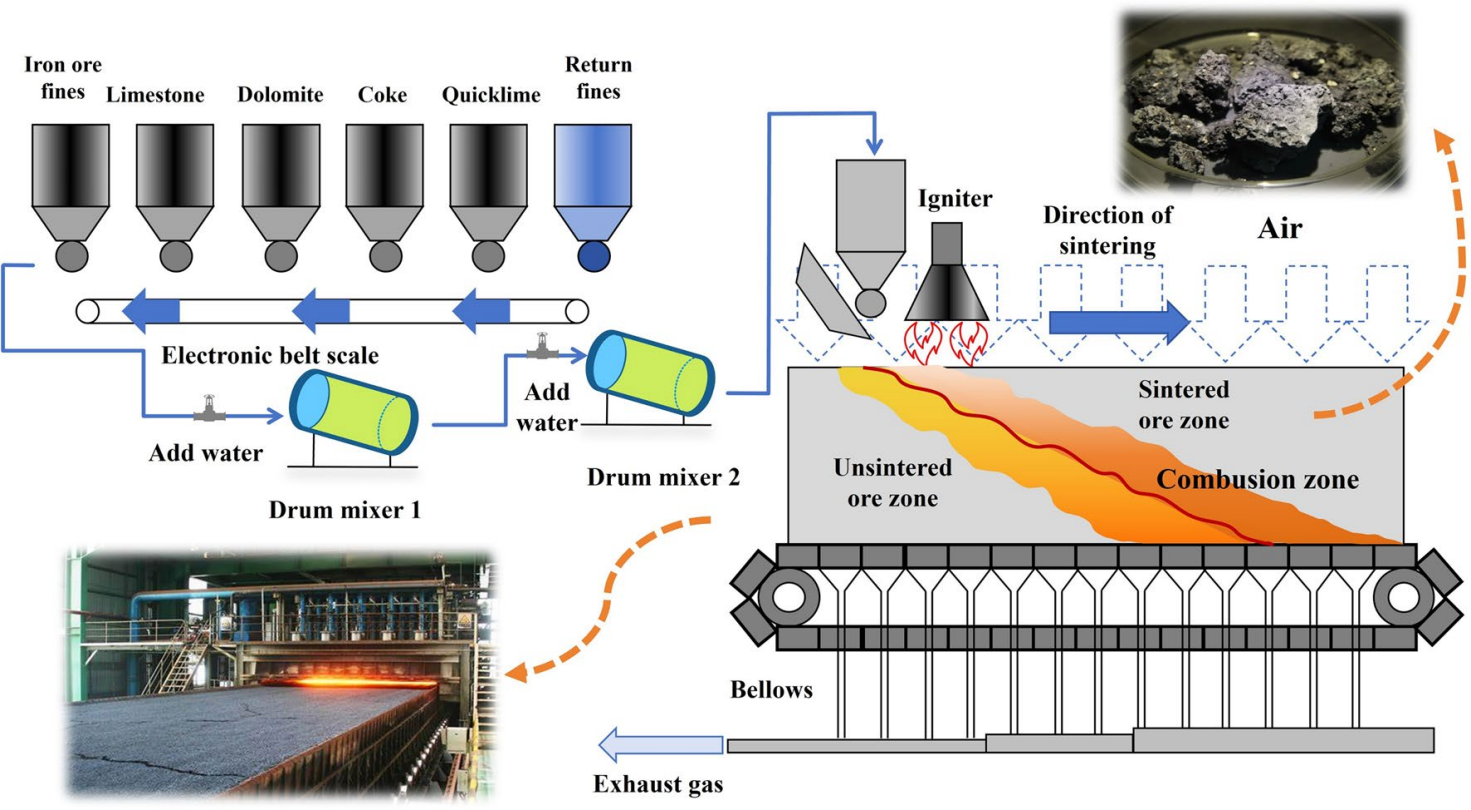
Sintering process flow.

Sintering is a critical stage in the blast furnace ironmaking process, ensuring smooth operation of the overall system. The primary objective of sintering is to transform mixed raw materials into iron ore by subjecting them to high-temperature treatment. The temperature distribution at each point inside the iron ore during the sintering process is the focus of the research and prediction in this study. High-quality iron ore exhibits excellent reducibility, permeability, and strength, which can significantly improve blast furnace efficiency while reducing energy consumption and cost. The sintering process can be broken down into several key stages, including raw material preparation, mixture mixing, ignition, and high-temperature sintering. (See Fig. 1 for details):

### Mathematical mechanisms

**Fig. 2:**
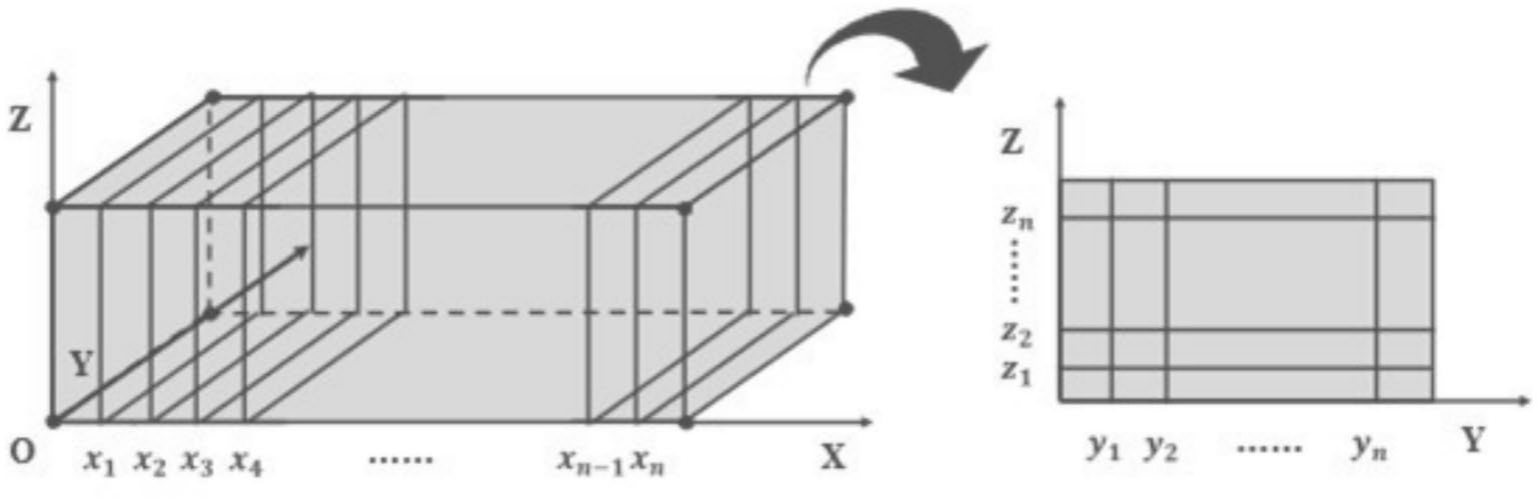
3D spatial and sliced coordinate system model of sintered ore.

To facilitate research and subsequent explanation of the research process and results, the mixture on the sintering machine pallet is approximately abstracted as a rectangular cuboid. Taking point O as the origin, the direction of the pallet’s movement, width, and height are defined as the positive X, Y, and Z axes, respectively, establishing a three-dimensional rectangular coordinate system. The coordinate ranges for all three dimensions are standardized as [0, 1]. The 3D diagram is shown in the left part of Fig. 2:

Next, the cuboid is discretized in a 3D grid ^17^, with the slices shown in the right part of Fig. 2. For each *x* value $x_1,x_2,\ldots ,x_n$ the “slice” with negligible thickness is extracted. For each slice, the temperature distribution is discretized into a matrix of $y_n \times z_n$, allowing a detailed study of the temperature distribution along the *y* and *z* axes during sintering. Finally, by summarizing the different slices, the approximate temperature can be obtained at each point (*x*, *y*, *z*) on the sintering pallet, representing the three-dimensional temperature distribution of iron ore.

### Physical mechanisms

Sintering involves the softening and partial melting of raw material particles, such as iron ore, at elevated temperatures. This process generates a liquid phase that interacts with unmelted ore particles. After being cooled, the liquid phase stores the mineral powder particles in blocks. Obviously, the sintering process is a block-making process involving high-temperature physical and chemical reactions.

Therefore, after abstracting the real sintering process into a mathematical model, this study proceeds to explain the temperature changes during the sintering process from a physical perspective.

- Solid-gas phase heat conduction equation
  During the sintering process, heat transfer inevitably occurs between the mixture and the gas, resulting in temperature differences and internal heat exchange between the solid and gas phases within the same space. Therefore, this study adopts a local non-thermodynamic equilibrium dual energy equation to characterize the variation of solid-phase temperature, $T_{s} = T_{s}(y,z,t)$, and gas-phase temperature, $T_{f} = T_{f}(y,z,t)$. Considering that in the previously established model the entire sinter bed was discretized along the *x*-direction into slices denoted by $x_i$, with each slice containing both the *y* and *z* dimensions, the one-dimensional energy equations for the solid and gas phases are extended into three dimensions. This allows for a more accurate simulation of heat transfer within the sintering bed and better reflects spatial temperature variations. It should be noted that although heat transfer between slices in the *x*-direction does exist, its effect is relatively small compared with those in the *y*- and *z*-directions. This is because the sinter bed has already been discretized along the *x*-direction in the modeling framework, and the sintering process is strongly influenced by external factors such as the induced draft fan located beneath the bed, which enhances vertical heat transfer.
  The solid-phase energy equation describes the energy changes within the solid phase, primarily including the conductive heat transfer within the solid, the heat source term, and the convective heat transfer between the solid and gas phases:
  1 $$\begin{aligned} & (1 - \phi )\rho _{s} c_{s} \frac{{\partial T_{{s,i}} }}{{\partial t}} \\ = & (1 - \phi )\left( {\frac{\partial }{{\partial y}}\left( {\lambda _{s} \frac{{\partial T_{{s,i}} }}{{\partial y}}} \right) + \frac{\partial }{{\partial z}}\left( {\lambda _{s} \frac{{\partial T_{{s,i}} }}{{\partial z}}} \right) + \frac{{\lambda _{s} }}{{\Delta x^{2} }}(T_{{s,i + 1}} - 2T_{{s,i}} + T_{{s,i - 1}} } \right) \\ & + (1 - \phi )q_{s} - h_{v} (T_{{s,i}} - T_{{f,i}} ) + Q_{s} ,\;(y,z) \in D,i = 1,...,n \\ \end{aligned}$$
  The gas-phase energy equation describes the energy changes within the gas phase, primarily including the convective and conductive heat transfer in the gas, the heat source term, and the convective heat transfer between the gas and solid phases:
  2 $$\begin{aligned} \begin{aligned}&\phi \rho _f c_f \frac{\partial T_{f,i}}{\partial t} + \frac{\partial }{\partial y} (\rho _f c_f u_{fy} T_{f,i}) + \frac{\partial }{\partial z} (\rho _f c_f u_{fz} T_{f,i}) \\&= \phi \left( \frac{\partial }{\partial y} \left( \lambda _f \frac{\partial T_{f,i}}{\partial y} \right) + \frac{\partial }{\partial z} \left( \lambda _f \frac{\partial T_{f,i}}{\partial z} \right) +\frac{\lambda _f}{\Delta x^2}\big (T_{f,i+1}-2T_{f,i}+T_{f,i-1}\right) +\\ &\frac{\rho _f c_f u_{fx} (T_{f,i}-T_{f,i-1})}{\Delta x} +\phi q_f - h_v (T_{s,i} - T_{f,i}) + Q_f,\quad (y,z) \in D, i= 1,...,n \end{aligned} \end{aligned}$$
  In the equation: $D= [0,1]\times [0,1]$; $\phi$ represents the porosity, indicating the volume ratio between the solid and gas phases; $\rho$ is the density; *c* is the specific heat capacity; $\lambda$ is the thermal conductivity; *q* is the internal heat source per unit volume; $h_v$ represents the volumetric heat transfer coefficient; $Q_s$ corresponds to the solid-phase reaction heat source; $Q_f$ is the gas phase reaction heat source; $u_{fy}$ and $u_{fz}$ is the velocity component of the gas. The value of $h_v$ can be calculated using Achenbach’s criterion formula^19^.
- Equation of conservation of heat energy
  Due to heat exchange between the high-temperature sintering mixture at the boundary and the cooler air during sintering, this study incorporates and analyzes the energy conservation equation. The thermal energy transfer in the fluid is characterized by three mechanisms in Eqs. (3)–(4)^18^: temporal variation, convection, and heat conduction.
  3 $$\begin{aligned} & \rho C_a \; \frac{\partial M}{\partial t} + \rho C_a \, u \cdot \nabla M + \nabla \cdot w = Q + Q_{ted} \end{aligned}$$
  4 $$\begin{aligned} & w = -k \, \nabla M \end{aligned}$$
  In the equation: $\rho$ is the density of the substance; $C_a$ is the specific heat capacity; *M* is the temperature; *u* is the velocity vector of the fluid; $\nabla M$ is the temperature gradient; *w* is the heat flux density vector; *k* is the thermal conductivity; *Q* is the volumetric heat source; $Q_{ted}$ represents other forms of heat sources or heat dissipation.

### Chemical mechanisms

Based on the mass and volume proportions of the chemical components in the sintering raw materials, as well as factors such as the heat of chemical reactions, the nature of heat absorption and release during chemical reactions, and the reaction rates, this study comprehensively considers these factors and identifies six key chemical reactions that significantly impact the sintering process. These reactions are analyzed in detail, as shown in Table 1:

**Table 1:** Summary of important chemical reactions.

Chemical reaction	Reaction equations	$\quad \Delta$H(KJ/mol)
*C* complete combustion	$2\text {C} + \text {O}_2 \rightarrow 2\text {CO}$	−393.5
*C* incomplete combustion	$2\text {CO} + \text {O}_2 \rightarrow 2CO_2$	−566.00
$CaCO_3$ decomposition	$\text {CaCO}_3(s) \rightarrow \text {CaO}(s) + \text {CO}_2(g)$	+179.5
$CaMg(CO_3)_2$ decomposition	$CaMg(CO_3)_2 \rightarrow CaO+\text {MgO} + 2\text {CO}_2$	+116.7
$Fe_3O_4$ oxidation	$4Fe_3O_4 + \text {O}_2 \rightarrow 6\text {Fe}_2\text {O}_3$	−1118.38
$H_2O$ gasification	$H_2O(l) \rightarrow H_2O(g)$	+44.01

The mechanism of the influence of the above chemical reactions is analyzed as follows. The complete and incomplete combustion of *C* acts as primary exothermic sources, continuously providing heat to the system. Although the decomposition reactions of $CaCO_3$ and $CaMg(CO_3)_2$ are endothermic processes, their products *CaO* and *MgO* combine with silicates to form minerals at low melting points, promoting the formation of liquid phases. Simultaneously, the released $CO_2$ optimizes the permeability of the sintering bed. Oxidation of $Fe_3O_4$ maintains a high temperature environment through exothermic effects, yet requires controlled reaction rates to prevent excessive melting. The endothermic gasification of $H_2O$ may lead to localized cooling, which requires a balanced moisture content to ensure permeability and uniformity of the sintering.

## Methods

To address the problem of predicting the internal spatial temperature of iron ore, the theoretical methods in this study include: the Variational Autoencoder- Temporal Convolutional Networks (VAE-TCN) 3D prediction model for the main chemical component proportions of the sintering mixture, and the Temporal Fusion Transformer (TFT) 3D prediction model for the temperature of iron ore. In this work, the focus is not on proposing a new TFT architecture, but on demonstrating its rational, effective, and innovative application to 3D spatial temperature prediction of the sintered ore, which has not been reported in previous studies.

### VAE-TCN

**Fig. 3:**
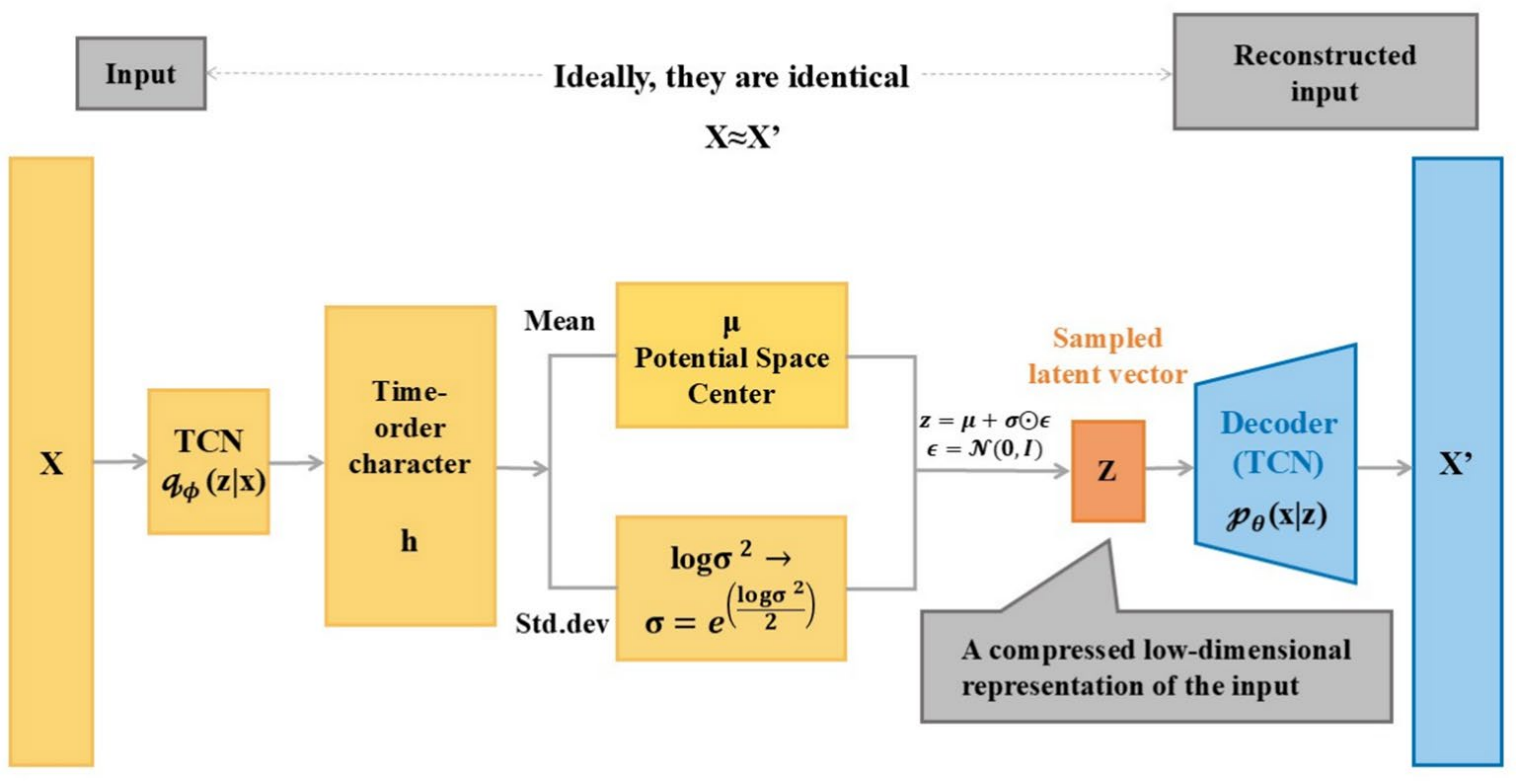
VAE-TCN model architecture.

The VAE-TCN model (Fig. 3) combines a Variational Autoencoder (VAE) and Temporal Convolutional Networks (TCN) to predict the main chemical composition ratios of the sintering mixture. TCN serves as both the encoder and decoder of the VAE. Its dilated convolutions capture long-term dependencies in time series, while the VAE’s latent space regularizes feature distributions through variational inference^20^.

#### Encoder and latent space

The encoder maps the input sequences to the mean $\mu$ and variance $\sigma$ latent distribution parameters, and generates latent variables *z* by reparameterization:

5 $$\begin{aligned} z = \mu + \sigma \odot \epsilon \end{aligned}$$

In the equation:*z* is a latent variable, representing low-dimensional features of the input data; $\mu$ is the mean of the latent variable distribution; $\sigma$ is a variance of the latent variable distribution; $\epsilon \sim \mathcal {N}(0,I)$ injects Gaussian noise for gradient propagation; $\odot$ denotes element-by-element multiplication.

#### Decoder and reconstruction

The latent variable *z* is decoded by TCN to reconstruct the sequence $\hat{x}$. Dilated convolutions (Eq.7) expand the receptive field exponentially:

6 $$\begin{aligned} \hat{x}= & \text {TCN} - \text {Decoder}(z) \end{aligned}$$

7 $$\begin{aligned} \hat{y}_t= & \sum _{i=0}^k w_i \cdot z_{t-d \cdot i} \end{aligned}$$

In the equation: $\hat{x}$ denotes the reconstructed output sequence, *z*is a latent variable, $\hat{y}_t$ is the output predicted at the time step *t*, $w_i$ is the weight of the i-th filter, *d* is the expansion factor, *k* is the filter size, and $t-d \cdot i$ is the direction of past development.

#### Joint loss function

The total loss combines VAE reconstruction loss, KL divergence, and TCN prediction error:

8 $$\begin{aligned} L_\textrm{total} = L_\textrm{VAE} + \lambda L_\textrm{TCN} = \frac{1}{N}\sum _{i=1}^N (x_i - \hat{x}_i)^2 - \frac{\beta }{N}\sum _{i=1}^N \biggl (\log \left( \sigma _i^2\right) + \frac{(z_i - \mu _i)^2}{\sigma _i^2} - 1\biggr ) + \frac{\lambda }{N}\sum _{i=1}^N \bigl (y_i - \hat{y}_i\bigr )^2 \end{aligned}$$

In the equation: $\lambda$ is the balance coefficient, $\beta$ is the weight hyperparameter, $y_i$ is the i-th actual value in the time series, $\hat{y}_i$ is the i-th predicted value in the time series, $x_i$ is the i-th sample in the original data, $\hat{x}_i$ is the i-th sample reconstructed by the decoder, *N* is the total number of samples, $\mu _i$ and $\sigma _i^2$ represent the mean and variance of the i-th latent space sample, respectively.

#### Residual connections

**Fig. 4:**
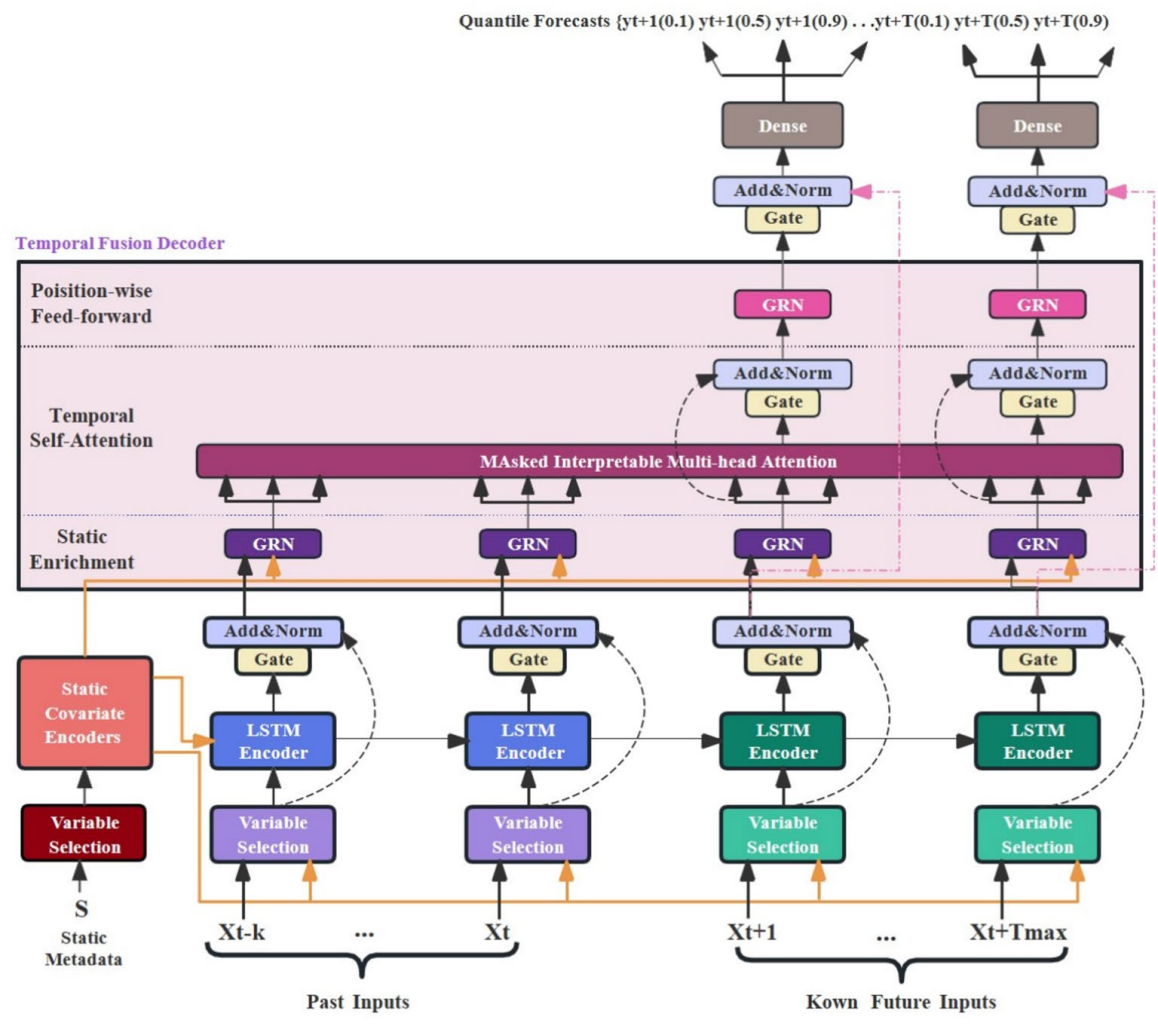
TFT model architecture. Adapted from the standard TFT architecture^16^.

Introduce skip connections (Fig. 4) to mitigate deep network degradation:

9 $$\begin{aligned} H(x) = F(x) + x \end{aligned}$$

In the equation: *H*(*x*) is the output of the residual block, *F*(*x*) is a residual function, representing convolutional layers and non-linear transformations.

### TFT

The Temporal Fusion Transformer (TFT) is an advanced architecture for interpretable multi-horizon forecasting, which integrates gating mechanisms, variable selection, attention mechanisms, and a sequence decoder to capture complex temporal dependencies^16^. The total architecture of the TFT is shown in Fig. 4

#### Gated residual network (GRN)

GRNs form the backbone of TFT, allowing the model to flexibly transform inputs while mitigating gradient vanishing or exploding problems. By incorporating gating layers, GRNs ensure that only relevant information is passed forward, enhancing model stability and efficiency.

#### Variable selection network (VSN)

VSNs operate at both static and temporal levels to automatically identify the most important variables at each step. This mechanism not only reduces redundancy but also enhances interpretability by highlighting the key factors influencing prediction.

#### Multi-head attention mechanism

The attention layer is designed to capture long-range temporal dependencies by focusing on the most relevant past information. Multi-head attention improves robustness by jointly attending to different aspects of the historical data.

#### Temporal fusion decoder

This module integrates local short-term patterns with long-term trends extracted by attention, enabling the model to generate accurate multi-step forecasts. The decoder structure fuses multiple information streams to produce the final output.

#### Loss function

TFT jointly optimizes multi-quantile prediction errors for robustness. The loss function enables the model to generate predictions at varying confidence levels, effectively adapting to random fluctuations in the sintering process.

10 $$\begin{aligned} L = \sum _{q \in \langle 0,1,0.5,0.9 \rangle } \sum _{t=1}^T \max \bigl (q\bigl (y_t - \hat{y}_t^{(q)}\bigr ), (q-1)\,\bigl (y_t - \hat{y}_t^{(q)}\bigr )\bigr ) \end{aligned}$$

In the equation: *q*is the quantile, $y_t$is the ground truth at the time step *t*, $\hat{y}_t^{(q)}$ is the quantile prediction at time step *t*, *T* is the total number of time steps.

## Results

**Fig. 5:**
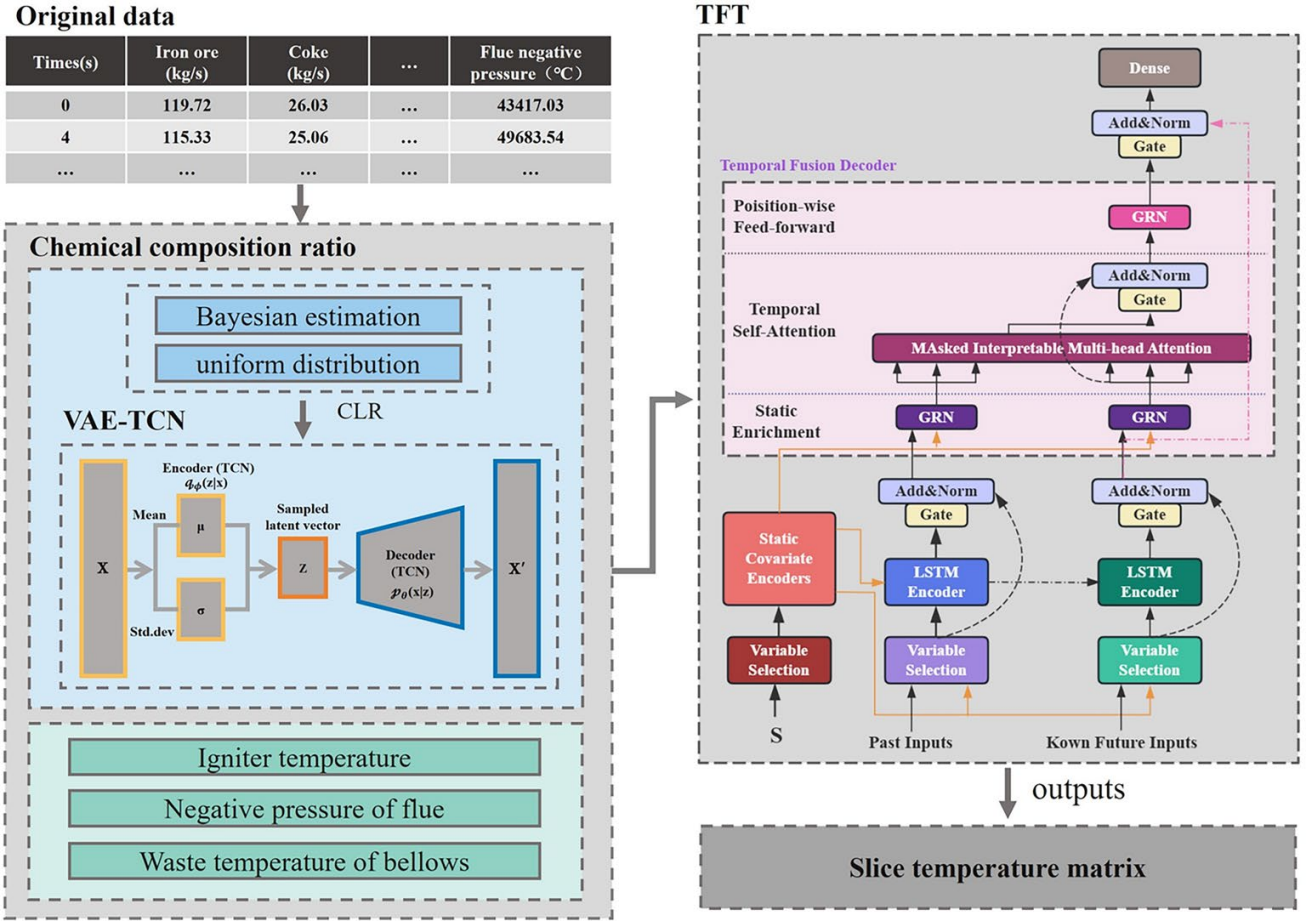
3D Temperature Prediction Model Architecture for Iron Ore Sintering.

As shown in Fig. 5, this study first performs data augmentation based on Bayesian estimation and uniform distribution, followed by a Centered Log-Ratio (CLR) transformation on the composition ratio data. The VAE-TCN model, suitable for compositional time series data, is used to obtain the proportion data of the main chemical components of the mixture. In addition to ignition temperature, flue negative pressure and windbox waste temperature, these are used as input variables for the TFT model, while the 2D temperature matrix of different slices is the output variable for training. Afterward, the trained TFT model can be used to predict the 3D temperature of iron ore based on the newly collected input variable data.

### Slicing data expansion

The experimental data in this study are sourced from the database of a steel plant in China (with a measurement accuracy of 0.01 for the distribution of sintering raw material and 0.01 for sensor variable measurements). The sintering data are mainly divided into sensor data and sintering chemical composition data. Sensor data parameters can be categorized into the following: Raw Material Parameters (RMP), Operating Parameters (OP) and State Parameters (SP). The parameters of the chemical composition of the sintering data parameters (CCP) are predicted based on the quantity of raw material distribution (as shown in Table 2).

**Table 2:** Summary of parameters of the sintering process dataset.

Category	No.	Parameters and units	No.	Parameters and units
RMP	1	Iron ore(kg/s)	2	Coke(kg/s)
	3	Dolomite(kg/s)	4	Limestone(kg/s)
	5	Quicklime(kg/s)	6	Pulverized coal(kg/s)
	7	Sintering return(t/h)	8	Blast furnace return(t/h)
OP	9	Round roll speed(r/h)	10	Nine-roller speed(r/h)
	11	Sintering machine speed(r/h)	12	Ignition temperature($^{\circ }$C)
	13	Gas flow(m3/h)	14	Fan air volume(m3/h)
SP	15	South flue temperature ($^{\circ }$C)	16	South flue pressure (kpa)
	17	North flue temperature ($^{\circ }$C)	18	North flue pressure (kpa)
	19-32	Bellows gas temperature ($^{\circ }$C)	33-46	Bellows negative pressure (kpa)
CCP	47	*TFe*(%)	48	*C*(%)
	49	$SiO_2$(%)	50	*CaO*(%)
	51	$Al_2O_3$(%)	52	*MgO*(%)
	53	Moisture content (%)	–	–

The specific measurement data used in this study were collected from the factory’s real-time monitoring system, covering the period from January 14, 2021, to September 1, 2021, with a sampling rate of 4 times per second. A total of 80,641 samples were collected (as shown in Table 3). Since the sintering process may be affected by environmental disturbances, sensor instability, and other perturbing factors, the raw data was pre-processed including missing data identification, outlier detection, and data normalization to improve the prediction performance of the subsequent model.

**Table 3:** Examples of historical data used in the experiment.

Times(s)	Iron ore(kg/s)	Coke(kg/s)	...	Igniter temperature($^{\circ }$C)	Flue negative pressure($^{\circ }$C)
0	119.72	26.03		1118.56	43417.03
4	115.33	25.06		1119.69	49683.54
8	120.32	23.77		1122.56	40140.52
12	121.35	25.01		1126.13	45670.26
16	116.08	23.08		1129.94	51568.55
**...**	**...**	**...**	**...**	**...**	**...**

For the measurement of the internal temperature of iron ore, this study focuses on analyzing the impact of the proportion of key chemical components in the mixture, including *TFe*,$SiO_2$,*CaO*,*MgO*,$Al_2O_3$ and *C* , as their fluctuations significantly affect the mineral composition and internal temperature variations of sintered ore. In addition to these, the study also considers other factors influencing the temperature of ignition, the negative pressure of the furnace, the waste temperature of the wind box, and the temperature of the sinter ore slices, all of which are sensor variables.

Due to the limited data on the content of the main chemical components in different raw materials, this study adopts Bayesian estimation and uniform distribution to reasonably augment the initial sample data in order to complete the comprehensive prediction of the proportion of the main chemical components in the sintering raw materials, based on the characteristics of the different datasets.

In Bayesian estimation, an inverse gamma distribution is set as the prior distribution for the unknown variance. Subsequently, based on the results of the K-S test, the K-S statistic for the component *TFe* in iron ore is 0.028395, with $p=0.5296>\alpha =0.05$, indicating that there is no significant difference between the sample data distribution and the theoretical distribution; therefore, it can be considered normally distributed. Since the original sample size is 120 (>100), the K-S test in this study has sufficient statistical power, and the obtained normality conclusion is considered reliable. Using Bayes’ theorem, the posterior distribution of the variance is updated, following the same principle. Ultimately, the proportion of each major chemical component in the sintering raw materials is approximated by a specific distribution. For example, the chemical composition of *TFe* in iron ore is approximately distributed as:*N*(62.1506, 12.4056) . For other components with lower contents and limited fluctuation, their proportions are approximately estimated to follow a uniform distribution. The distribution data for the main chemical components of iron ore are shown in Table 4.

**Table 4:** Iron ore content of major chemical components as a percentage of obedience distribution.

Chemical components	Components	Chemical components	Components
*TFe*	N(62.15,12.41)	$SiO_2$	N(4.70,4.84)
*MgO*	U(0.10,0.20)	*CaO*	U(0.08,0.23)
$Al_2O_3$	U(1.23,3.11)	*C*	U(0,0)

Considering that the proportion data of components have a sum constraint, which induces compositionality, this study applies the Centered Log-Ratio (CLR) transformation to map the chemical composition proportion data into the real number space. This transformation eliminates the sum constraint while preserving the relative relationships among the data of the original component, thereby improving the analyzability of the data and improving the accuracy and robustness of the subsequent VAE-TCN prediction model.

### Analysis of VAE-TCN training prediction

Using component proportion data derived from Bayesian estimation and uniform distribution, this study applies a VAE-TCN combined model to analyze internal data relationships and predict the real-time dynamics of the mixture’s main chemical component proportions. When training the VAE-TCN prediction model, 75% of the data set is used for training samples and 25% for testing samples. To further ensure the integrity of the results, the paper also performed five additional 10-fold cross-validation runs on the training set. The final model parameters are shown in Table 5.

**Table 5:** Model parameters.

Model parameters	Value
Input_shape	(7, 6)
Latent_dim	2
Filters1	12
Filters2	6
Kernel_size	3

The input dimension of the VAE-TCN model is (6, 7), where 6 represents the number of time steps and 7 corresponds to the number of feature variables. This clarification ensures that the temporal structure and multivariate characteristics of the data are explicitly captured in the modeling process. Compared to the previous layer Filters1, the number of convolutional kernels in Filters2 is reduced, helping to reduce model parameters and lower the risk of overfitting, while still capturing important features. Overall, these two convolutional layers process the input data sequentially, first extracting features using 12 convolutional kernels, and then further processing these features with 6 convolutional kernels.

The padding strategy is configured as ”the same,” ensuring that the output sequence length matches the input sequence after convolution. This is achieved by adding appropriate zero padding to both sides of the input data. The model consists of convolutional layers, a Flatten layer, Dense layers, Lambda layers, a Repeat Vector layer, and GRU layers, with a total of 1125 trainable parameters. Table 6 presents the model’s training and testing performance metrics. The model underwent 50 training epochs using mean squared error as the loss function. Both training and validation losses gradually decrease and converge, demonstrating the model’s resistance to overfitting and strong generalization capability, making it suitable for accurately predicting the chemical composition ratios.

**Table 6:** Indicators for assessing the results of the model.

Epoch	loss	val_loss
1	0.9570	0.9664
10	0.1674	0.1542
20	0.0618	0.0722
30	0.0151	0.0163
40	0.0047	0.0046
50	0.0012	0.0013

**Fig. 6:**
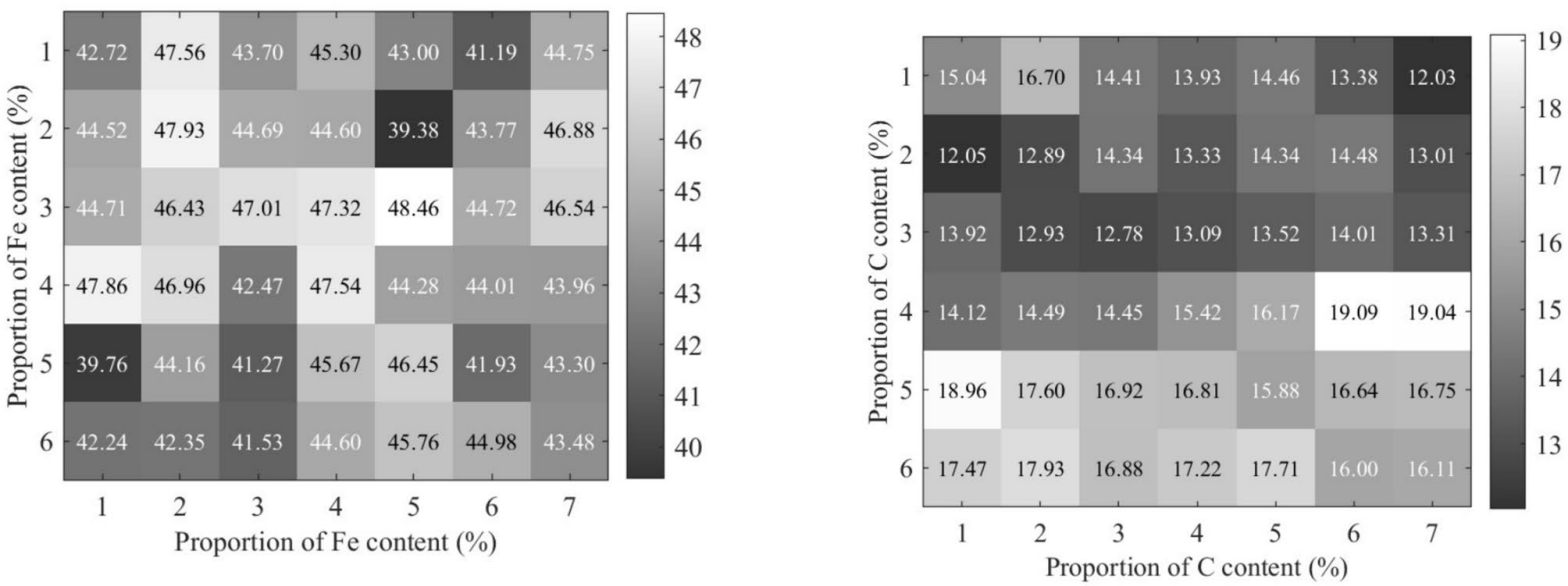
Example of data matrix for *TFe* and *C* content share.

To predict the chemical composition ratios of the sinter mixture using the trained VAE-TCN model, a 2D data matrix is obtained, as shown in Fig. 6.

### Analysis of TFT training

The TFT sinter temperature spatial prediction model was trained using the major chemical composition ratio data predicted by the VAE-TCN model and sensor variable data. The dataset was partitioned with 90% allocated for training, 5% for validation, and 5% for testing purposes. Hyperparameter optimization is performed on the training set through grid search combined with 5 runs of 10-fold cross-validation to ensure robustness and prevent overfitting. Table 7 reports the final hyperparameter settings for one representative slice used in this study. Due to space limitations, only the hyperparameters of this slice are presented as an example.

**Table 7:** Model Parameters.

Model Parameters	Value
Learning rate	0.001
Dropout rate	0.2
L2 regularization coefficient	0.001
Number of attention heads	4
Hidden dimensions	160
Number of single batches	64

As shown in Table 8, the root mean square error (RMSE) and the relative root mean square error (RMSEr) between the actual values and the temperature matrices of different slices predicted by the TFT sinter temperature spatial prediction model are both relatively low, and the coefficient of determination ($R^2$) is close to 1. This indicates that the model can accurately and reasonably predict the internal temperature of the sinter during the sintering process.

**Table 8:** Evaluation metrics of the model results.

Slice	RMSE	RMSEr	$R^2$
1	4.7507	0.0185	0.8756
2	4.7618	0.0171	0.8375
3	4.7636	0.0171	0.8562
4	4.7624	0.0171	0.8245
5	4.7506	0.0185	0.8737
6	4.7514	0.0185	0.8757

**Fig. 7:**
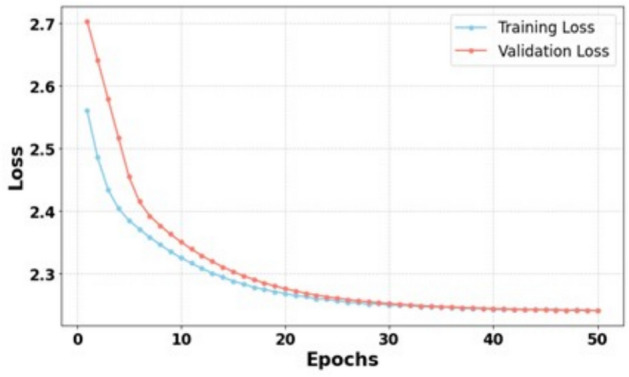
Plot of training validation loss results.

As shown in Fig. 7, the training loss and validation loss of the TFT model decrease rapidly during the early stages of training and gradually stabilize. The validation loss fluctuates minimally within a certain range, indicating that the model has good convergence and can effectively capture both short- and long-term dependencies in time series data. The close alignment between the training and validation loss curves further validates that the model is robust, making it suitable for handling noisy time-series data and capturing key features.

To further evaluate the TFT model’s capability in predicting sintered ore internal temperature, this study evaluates its performance against alternative time series models capable of handling diverse input variables. Recent years have witnessed extensive applications of LSTM models in time series forecasting due to their effective long-term dependency capture through gating mechanisms. Building upon this foundation, the TFT architecture enhances the original Transformer framework by combining its powerful parallel processing capacity with improved global information extraction capabilities. Therefore, this study selects these two models, LSTM and Transformer, and compares their prediction results with those of the TFT model.

As shown in Table 9, differences in the coefficient of determination ($R^2$), the mean square error (RMSE), and the relative mean square error (RMSEr) between the three models were analyzed. The comparison reveals that the TFT model achieved lower RMSE and RMSEr compared to the LSTM and Transformer models, with $R^2$ improving to 0.8572. These results demonstrate significant improvement in the TFT model’s predictive performance, with the prediction results much closer to the target values, indicating a good prediction effect.

**Table 9:** Comparison of the values of the indicators for assessing the performance of different models.

Model	RMSE	RMSEr	$R^2$
LSTM	7.7505	0.0682	0.7279
Transformer	5.5618	0.0385	0.7975
TFT	4.7568	0.0178	0.8572

### Analysis of TFT forecast results

The TFT model enhances the Transformer model’s performance by quantifying attention weights within the variable selection hierarchy. This enables the analysis of input feature importance on the output variable, specifically the sinter ore temperature matrix prediction. In doing so, the TFT model provides valuable information for factory control systems, guiding the monitoring and adjustment of key features to improve the accuracy and efficiency of the sintering process.

As shown in Table 10, the igniter temperature plays a key role in determining the temperature of the sinter ore slice. Since the igniter temperature directly affects the temperature of the mixture, it is crucial to control the accuracy and stability of the igniter temperature. Among the main chemical components of the mixture, the content of *TFe* has the greatest impact on the temperature of the slicing. *TFe* , being a core component of steel materials, aligns with the findings of this experiment, which are consistent with practical observations.

**Table 10:** Importance of features.

Input features	Significance	Input features	Significance
*TFe*	0.1204	*C*	0.1110
$SiO_2$	0.1112	Flue negative pressure	0.1163
*CaO*	0.1071	Ignition temperature	0.1450
*MgO*	0.1069	Windbox waste temperature	0.0778
$Al_2O_3$	0.1044		

**Fig. 8:**
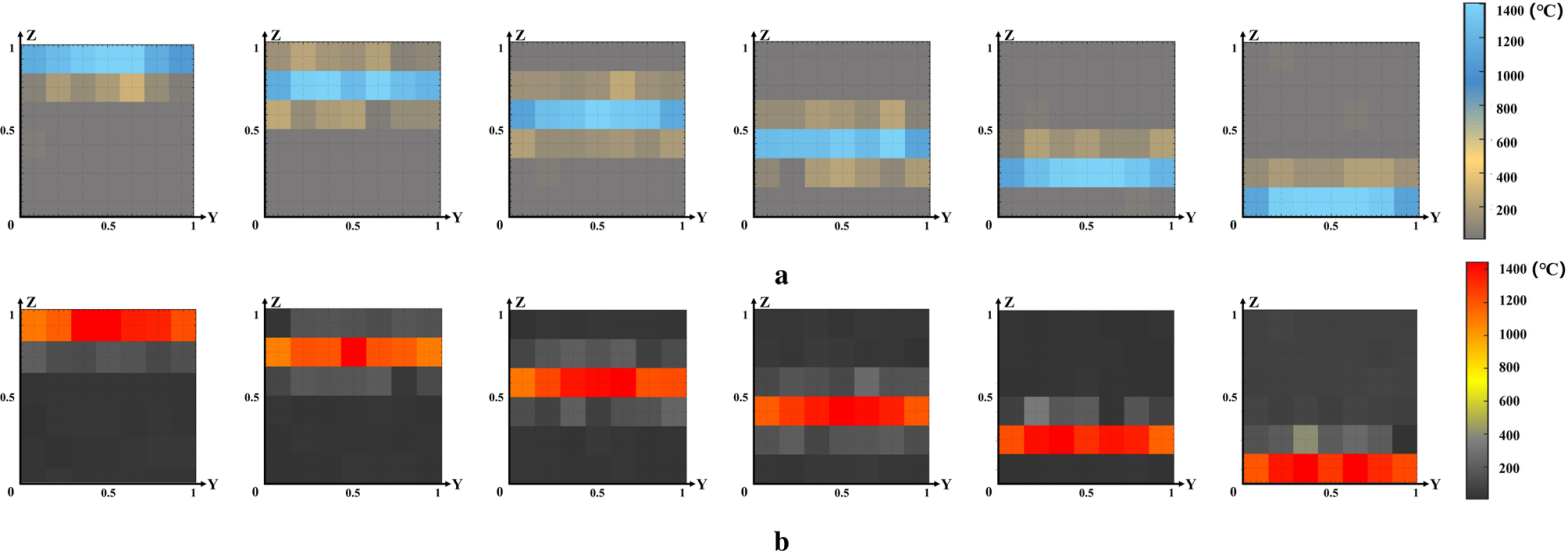
Slicing temperature. At the same input data at a certain time, the heat map of the predicted value of the temperature of the six slices (**a**) and the actual value (**b**) of the sinter at the same position.

As shown in Fig. 8, for the specific prediction results of the TFT model on the two-dimensional temperature matrix of sinter slices, the predicted value (Fig. 8a) and the actual value (Fig. 8b) of six slices at a certain time were selected for visualization and comparative analysis. The complete predicted temperature matrices and corresponding detailed numerical results are provided in the Supplementary Material for reference. As the x coordinate of the slice position increases, the blue area gradually shifts downward, reflecting the sintering process where the highest temperature reaction zone moves from the top to the bottom of the material. At the same time, the boundaries of the high-temperature zone of the slices at different positions showed obvious temperature drops, which were caused by heat exchange between the sinter and the external environment. Due to the significant temperature difference between the high-temperature region and the external environment, convection and heat conduction play a major role, aligning with the physical principles outlined in the mechanism. Moreover, comparing the actual temperature value with the predicted results, the temperature difference in the high temperature area is basically stable in the range of 3.5 % of the temperature in the region.

### Simulation results analysis

At present, in practical application, there are two common methods for image monitoring of the material surface of the sintering machine: one is to use the ordinary visible light camera to obtain an intuitive image, that is, the visible light camera is used to shoot the image recognition system of the tail section of the sintering machine, but it can only identify the high temperature image, and can’t make an accurate judgment on the medium and low temperature image, nor can it obtain all the information of the thermal state of the section; The second is to use the infrared thermal imaging camera device to obtain thermal images, to realize the online monitoring of the thermal images of the sintering machine ore seam section and the blockage of the sintering table at the bottom of the sintering machine.

To visually represent the spatial distribution of the internal temperature in the sintered ore during sintering, a 3D simulation was conducted using simulation software. Parameters matching those of the steel mill data source were used to replicate real sintering conditions, and the simulated temperature data were compared with the model predictions.

**Fig. 9:**
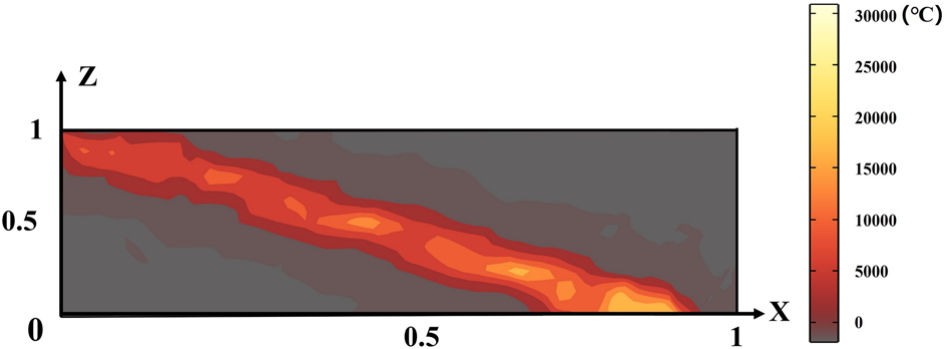
Simulation of y-axis slices of sintered ore.

On the one hand, the simulation of the sintered ore slices along the y-axis is shown in Fig. 9, showing the temperature field along the *x* and *z* axes. It can be observed that the high temperature region gradually moves in the negative direction of the z-axis as the coordinate value of the x-axis increases.

On the other hand, by selecting the simulation results for a specific moment of the sintered ore slice along the x-axis and comparing them with the results of the 3D sintered ore temperature prediction model established in this paper, the temperature field distribution along the *y* and *z* axes is observed to further analyze and validate the prediction performance of the model established in this paper.

**Fig. 10:**
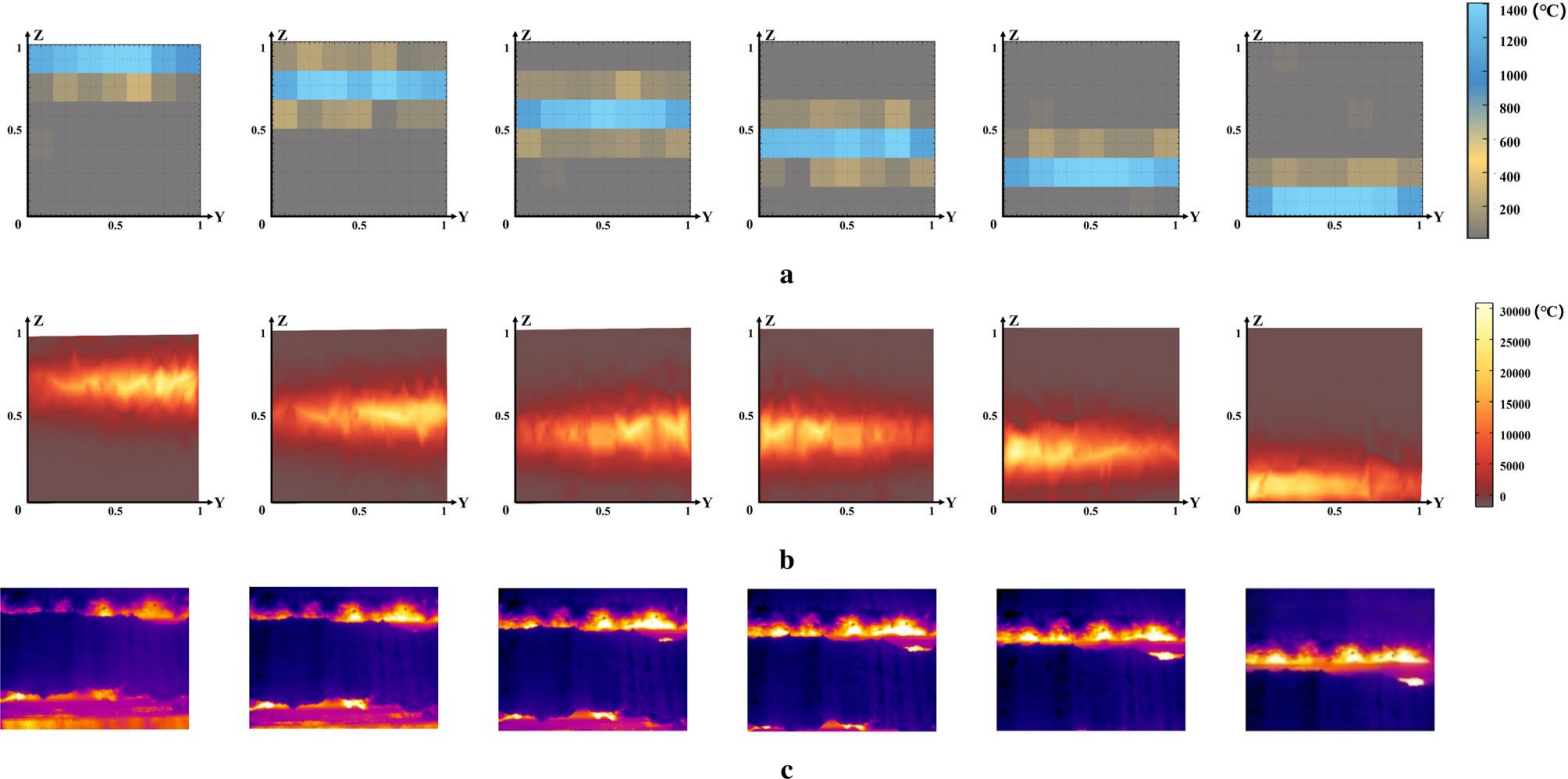
Comparison of sinter temperature model prediction (**a**), 3D simulation (**b**), and infrared thermal image (**c**)^22^. The three subfigures correspond to the same time slice of the sintering process, allowing a direct comparison of the predicted temperature field, the simulated temperature field, and the actual observed thermal image.

As shown in Fig. 10, the first row (Fig. 10a) shows a simulation of sinter temperature simulated by software, while the second row (Fig. 10b) shows a visualization of the heat map of sinter temperature based on the results of a simultaneous 3D prediction model, and the third row shows an example of an infrared thermal image pattern (Fig. 10c).

The comparison and analysis of model prediction map, simulation map, and infrared heat map are as follows:

- Based on the model predictions, simulations, and thermal images, three distinct zones can be clearly identified: the sintered, high temperature, and unsintered zones, which correspond to three different states of the sintered ore during the actual sintering process.
- According to the previously established 3D spatial coordinate system model of sinter temperature, the x-coordinates corresponding to the six slice positions are 1/6, 2/6, 3/6, 4/6, 5/6 and 1. The model predictions, simulations, and infrared heat maps all reflect roughly equal temperatures at different slice locations along the y-axis. However, because of factors such as chemical composition and boundary heat conduction, all three types of plot show some fluctuations. Simulation plots and infrared heat maps show these fluctuations in more detail, while model prediction plots provide a more general depiction of temperature fluctuations due to the specific grid method chosen in this study. However, the trends shown in both graphs are largely consistent and match actual observations, further confirming the strong predictive performance of the 3D sintered ore temperature prediction model developed in this study. It should be emphasized that the simulation results presented in this paper mainly serve as additional evidence to validate the effectiveness of applying deep learning models for 3D prediction of sintered ore temperature fields. For practical deployment, the preferred method is the learned deep model, as it offers higher efficiency, shorter computation time, lower cost, and competitive accuracy compared to finite element–based simulations.
- Looking at the model prediction plot, simulation plot, and infrared heat map along the z-axis, the high-temperature region gradually shifts from top to bottom as the x-coordinate of the slice increases, representing the progress and final completion of the sintering process, which is consistent with reality.

## Discussion and conclusion

This study develops a self-learning TFT model based on historical data, enabling more precise monitoring and control of sinter temperature throughout the sintering process. Additionally, through slice extraction and discretization of the sinter, the control range is successfully extended to three dimensions.

First, a 3D spatial coordinate system model was established for the sintering machine and the sintered ore. Next, the mechanism of temperature variation in the sintered ore during sintering was then analyzed. From a physical perspective, continuous heat exchange occurs between the sinter mixture and the gas. This study employs the dual-energy equation and the heat conservation equation under local non-thermodynamic equilibrium to provide a theoretical explanation; from the perspective of chemical reactions, the combustion process of the sintering material is accompanied by interactions and chemical reactions among the chemical components, which produce heat absorption and release. Six core chemical reactions were selected for the analysis of the mechanism. Next, due to limited chemical composition data across raw materials, this study applies Bayesian estimation or uniform distribution to reasonably augment the initial sample data, tailored to the characteristics of the dataset. After applying the CLR transformation, the VAE-TCN model, suitable for compositional time series data, was used to comprehensively predict the main chemical composition ratios in the sintering raw materials, resulting in a 2D composition ratio data matrix. The six main chemical composition ratio 2D data matrices, along with ignition temperature, flue gas negative pressure, and windbox waste temperature, were used as input variables, while the 2D temperature matrices of different slices were set as output variables and fed into the deep learning model TFT for training. The surface temperature data originate from factory-collected sensor measurements. However, since there are currently no practical sensors available to directly measure the internal temperature of the sintered bed and such data are difficult to obtain, the interior 2D temperature matrices employed in this study were derived from a combination of physicochemical mechanisms, simulation software, and partial real measurements. This not only ensures the availability of training data but also underscores the practical significance of this research in addressing the limitations of current industrial monitoring. The trained model can achieve 3D spatial predictions of sintered ore temperatures based on newly collected input variable data. Experimental results demonstrate the model’s superior performance compared to conventional time series approaches, with the mean $R^2$ of multistep predictions being 0.8572 and the RMSE being 4.7568. The model’s interpretability enables quantitative analysis of multiple factors affecting sintered ore temperature through its attention weight mechanism. Finally, simulation software was used to simulate the sinter temperature study process in 3D. Comparative analysis revealed strong agreement between the simulation results, TFT model predictions, and actual infrared thermal imaging data, thereby validating the model’s reliability.

Future research will optimize the model and integrate it with the best control algorithms, and by integrating with the factory automation system, key parameters, such as ignition temperature, can be dynamically adjusted, improving sintering stability and economic efficiency, while reducing energy consumption and carbon emissions. The model can also integrate with Industry 4.0 technologies, such as IoT and big data, to advance sintering intelligence. Additionally, it can be adapted to other high-temperature processes, including cement production and ceramic sintering.

## Supplementary Information

Supplementary Information.

## Data Availability

The data that support the findings of this study are available from the corresponding author upon reasonable request. Please contact Jiang Yushan at jys@neuq.edu.cn.
